# Modifying Dendritic Cell Activation with Plasmonic Nano Vectors

**DOI:** 10.1038/s41598-017-04459-1

**Published:** 2017-07-14

**Authors:** Kieng Bao Vang, Ingrid Safina, Emilie Darrigues, Dmitry Nedosekin, Zeid A. Nima, Waqar Majeed, Fumiya Watanabe, Ganesh Kannarpady, Rajshekhar A. Kore, Daniel Casciano, Vladimir P. Zharov, Robert J. Griffin, Ruud P. M. Dings, Alexandru S. Biris

**Affiliations:** 1 Center for Integrative Nanotechnology Sciences, University of Arkansas at Little Rock, 2801 S University Avenue, Little Rock, AR 72204 USA; 2 Department of Radiation Oncology, Winthrop P. Rockefeller Cancer Institute, University of Arkansas for Medical Sciences, 4301 West Markham Street, Little Rock, AR 72205 USA; 3 Arkansas Nanomedicine Center, Winthrop P. Rockefeller Cancer Institute, University of Arkansas for Medical Sciences, 4301 West Markham Street, Little Rock, AR 72205 USA

## Abstract

Dendritic cells (DCs) can acquire, process, and present antigens to T-cells to induce an immune response. For this reason, targeting cancer antigens to DCs in order to cause an immune response against cancer is an emerging area of nanomedicine that has the potential to redefine the way certain cancers are treated. The use of plasmonically active silver-coated gold nanorods (henceforth referred to as plasmonic nano vectors (PNVs)) as potential carriers for DC tumor vaccines has not been presented before. Effective carriers must be able to be phagocytized by DCs, present low toxicity, and induce the maturation of DCs—an early indication of an immune response. When we treated DCs with the PNVs, we found that the cell viability of DCs was unaffected, up to 200 μg/ml. Additionally, the PNVs associated with the DCs as they were phagocytized and they were found to reside within intracellular compartments such as endosomes. More importantly, the PNVs were able to induce expression of surface markers indicative of DC activation and maturation, i.e. CD40, CD86, and MHC class II. These results provide the first evidence that PNVs are promising carriers for DC-based vaccines and warrant further investigating for clinical use.

## Introduction

Dendritic cells (DCs) have the unique ability to capture, process, and present antigens to induce an adaptive immune response against viruses, pathogens, and cancer. In order to do this, DCs must be “educated” or “trained” so they can recognize the pathogen of question and start to initiate the process of an immune response. This education process involves turning on, or, up-regulating cell surface receptors on the DCs.

In general, DCs exist in two states: a resting state (immature phenotype) and an activated state (mature phenotype), with distinct cell surface receptor expression levels to indicate their specific state. Examples of these receptors are the major histocompatibility markers (MHC) class I, MHC class II, and their associated co-stimulatory markers known to be important in T-cell activation and stimulation: CD80, CD86, and CD40^1–3^. The markers, CD11c (alpha X integrin) and DEC-205 (a type I cell surface protein also known as CD205), are expressed by lymphoid DCs^4^. Altogether, these cell surface receptors work in concert to mobilize the adaptive immune response. Of importance is the MHC, which presents processed antigens in the form of peptides to the T cell receptor found on the surface of the T cells^5, 6^. This endows the T cells with the capacity to seek and eliminate the pathogen or, cancer of question. In the cancer field, this specialized DC function has been exploited to develop cancer vaccines^7–11^.

However, cancer has evolved sophisticated mechanisms to evade the immune response. Some of these mechanisms allow the cancer to induce an immune suppressive environment, thereby reducing the quality of the immune response, or to edit their cancer antigens so that the cells of the immune system are no longer able to detect and eliminate them^12–16^. In order to combat and overcome these immune suppressive mechanisms, at least in part, adjuvants are often included with vaccines to further stimulate the immune system^17–19^.

Several studies have investigated the use of nanoparticles (NPs) as cancer vaccine delivery vehicles. Ghotbi *et al*. assessed DC uptake of polyD L-lactide-co-glycolide (PLGA NPs) linked with mannan^20^. However, these studies did not present any information on the maturation state of the DCs^20^. Another report used carbon nanotubes (CNTs) as a delivery vehicle of the melanoma antigen NY-ESO-1. They showed that DCs were able to internalize the CNTs and that this provided protection against cancer^21^. Gold-based NPs (AuNPs) have major advantages over carbon-based and other nanomaterials (e.g., graphene, metal oxide, and polymeric NPs), including a low toxicity profile and FDA approval for clinical pilot studies^22–25^. Nevertheless, AuNPs have been shown to induce contradictory effects on DC maturation and their inflammatory state^26, 27^. For example, human-derived monocyte DCs treated with AuNPs, which were covered with glycomimetic DC-specific intercellular adhesion molecule-3-grabbing non-integrin ligands, were not activated, as evidenced by decreased expression of CD86^28^. In contrast, AuNPs coated with poly(diallyldimethyl ammonium chloride), or polyethylenimine, were used as a vaccine carrier for the HIV-1 Env plasmid DNA and was able to induce the maturation of both CD80 and CD86 on DCs^29^. However, the effect of surface plasmon, specifically plasmonically active silver-coated Au nanorods (AuNR/Ag), on DC activation and maturation has not been documented until now.

Our group has been developing a nanomaterial platform based on AuNRs with various coatings and functionalizations. One such platform is AuNR coated with a thin (1–2 nm) layer of Ag. These AuNR/Ag complexes provide advantages over basic AuNRs, as the Ag layer increases the surface-enhanced Raman Spectroscopy (SERS) signal approximately 129 times from that of AuNRs alone^30^. For the purpose of this paper, unless otherwise indicated, all experiments have been done with PEGylated (polyethylene glycol-covered) AuNR/Ag covered with the organic Raman molecule *p*-aminothiophenol (PATP); the resultant NPs are called plasmonic nano vectors (PNVs). In addition to their enhanced SERS signal, the PNVs have excellent photoacoustic (PA) and photothermal (PT) spectroscopic signatures, allowing for multimodal spectroscopic detection of them in complex biological systems^30^.

**Figure 1:**
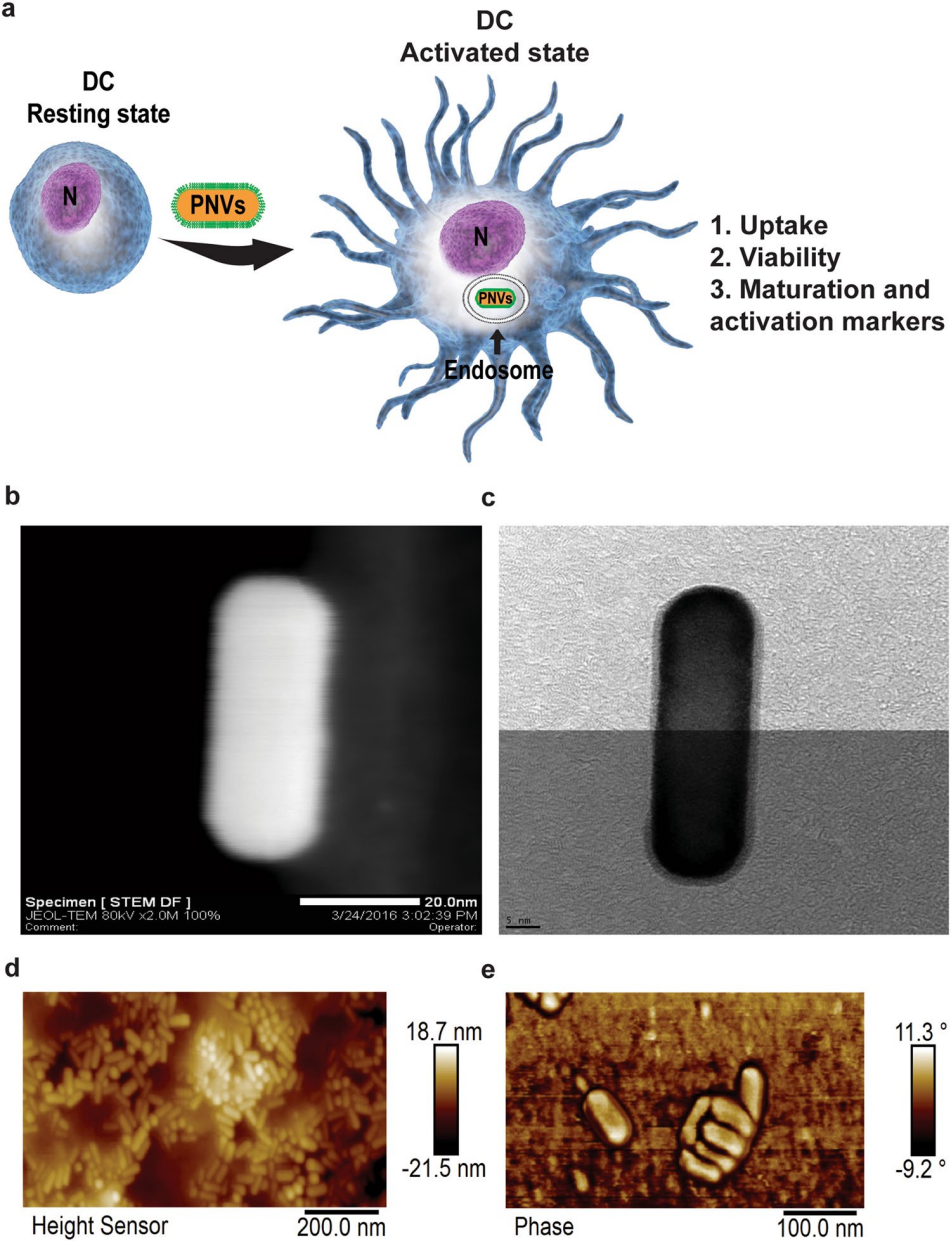
A schematic of the PNVs’ induced receptor changes on DCs and their dimensions. (**a**) Three factors can contribute to the functional state of DCs. (1) PNVs are engulfed by the DCs. (2) They have no effect on cell viability. (3) PNVs can induce up-regulation of activation and maturation markers on DCs. In the schematic, N = nucleus. The dimensions of PNVs coated with a layer of Ag. (**b**) STEM HAADF and (**c**) TEM image of PNVs showing the size of the AuNRs with a thin 1–2 nm layer of Ag. AFM images of the PNVs at different scanning scales as shown in (**d**), left side image is the scanned height view of the PNVs, and (**e**), right side image is the phase view of the PNVs. The AFM data is representative of 20 different images.

Three factors must be demonstrated in order to evaluate PNVs as potential carriers for DC-based cancer vaccines (Fig. 1a). First, the PNVs must be taken up by DCs. Second, the impact of PNVs on the cell viability must be minimal. Third, they should be able to induce DC receptors involved in activation and maturation. Altogether, these factors combined must be able to change the state of DCs from a resting state to an activated state.

Herein, we used the JAWSII DC line (henceforth termed as DC), which naturally exists in an immature state, also indicated by their low receptor expression levels of the aforementioned surface receptors and thus are an ideal cell line enabling detailed mechanistic studies^1–3^. We assessed the suitability of gold-based PNVs as DC delivery vectors. We found that PNVs were non-cytotoxic, were phagocytized by DCs, and they can induce DC activation and maturation. These features, along with the PNVs’ imaging capability, makes this vector an intriguing new platform for potential cancer vaccine delivery and treatment.

## Materials and Methods

AuNR/Ag was used as the baseline vector and covered with PATP, creating the PNVs^30^. Unless otherwise indicated, all the PNVs were subsequently PEGylated. Ninety percent of the prepared PNVs had an aspect ratio (AR) of approximately 3.0 ± 0.23^30^. For the confocal studies, the PNVs were conjugated to Green Fluorescent Protein (GFP, Novus Biologicals, NBC1-22949). Briefly, the HS-PEG-COOH functionalized SERS nano-agents were reacted with the N-terminals of GFP protein in the presence of N-hydroxysuccinimide (NHS) and N-(3-dimethylaminopropyl)-N′-ethylcarbodiimide hydrochloride (EDC). In this method, first a reactive ester of the carboxyl end of the SERS nano-agents is formed in the presence of NHS and EDC, which further reacts with the N-terminal of GFP (or any free amine group present in the protein) to furnish covalently linked (amide bond) GFP protein to the SERS nano-agents^31^. The resulting AuNR\Ag\PATP\PEG-GFP (PNV-GFP) were purified by centrifugation at 10,000 rpm twice. Finally, the PNV-GFP was re-dispersed in 1X PBS for further use^32^.﻿

### Cell Systems

#### JAWSII DCs

JAWSII DCs, an immortalized cell line derived from the bone marrow of *p53* ^*−/−*^ C57BL/6 mice (American Type Culture Collection (ATCC), CRl-11904), were grown in 10% fetal bovine serum (ATCC, 30-2020) and Alpha Minimum Essential Medium (Corning, Cat. 10-022-CV), 1% penicillin + streptomycin, and 5 ng/mL murine GM-CSF (R&D Systems, 415-ML-050, Minneapolis, Minnesota). Cells were maintained at 5% CO_2_, 37 °C, 100% humidity.

### Flow Cytometry

Cells were treated with the following concentrations of PNVs: medium alone and 1,10, and 50 μg/mL for 0, 3, and 7 days. Afterwards, the cells were washed and counted, and 1.0 × 10^6^ cells were stained at 4 °C for 30 minutes with the following antibodies (Affymetrix, eBiosciences): CD11c (N418), CD80 (16-10A1), CD86 (GL1), MHC Class I (3 4-1-2S), MHC Class II (M5/114/15/2), CD40 (IC10), and CD205 (205yekta). Subsequently, the cells were washed and fixed with 2% paraformaldehyde at 4 °C for 30 minutes, and flow cytometry was performed using an LSRFortessa (BD Biosciences, Franklin Lakes, NJ) at the Flow Cytometry Core at the University of Arkansas for Medical Sciences (Little Rock, AR), as described previously^33–36^. The data were analyzed using FlowJo software (TreeStar, Ashland, OR).

### Cell Viability

For viability studies, we used a WST-1 kit (Roche). DCs were seeded in 96-well plates at 10,000 cells/well and allowed to adhere overnight. Once attached, the DCs were treated with 1, 10, 100, and 200 μg/ml of AuNR/Ag or PNVs in a final volume of 100 µL and incubated for an additional 24 hours at 5% CO_2_, 37 °C, 100% humidity. After incubation, 10 µl of the WST-1 reagent was added to each well, and after 2 hours, the absorbance was read at 420–480 nm, similarly to how we have described before^37, 38^.

### Annexin V

Apoptosis was detected using the Annexin V Apoptosis Detection Kit (eBioscience). Cells were allowed to adhere over night and then were treated with medium alone or with AuNR/Ag at 1, 10, 25, and 50 μg/mL for 5 days. Afterwards, the cells were trypsinized and washed with 1X binding buffer, followed by staining with 5 μL of fluorochrome-conjugated Annexin V. After 15 minutes of incubation at room temperature, the cells were washed and resuspended, and propidium iodide was added according to the manufacturer’s recommendation.

### Atomic Force Microscope (AFM)

AFM was used to image the PNVs. The samples for AFM were prepared by dispensing the solvent (1X PBS) containing the PNVs (1000 μg/mL) on a silicon (Si) substrate at several spots. The Si substrate containing the PNV solvent was dried overnight under a chemical fume hood. This resulted in well dispersed PNVs that adhered to the surface of the substrate. The tapping mode of Bruker Fastscan AFM was utilized to scan the PNVs with a scan rate of 1 Hz and 256 samples per line. Both height and phase images were recorded during the scanning. The Bruker Nanoscope Analysis software (version 1.8) was used to polish the images.

### Raman Spectroscopy

**Figure 2:**
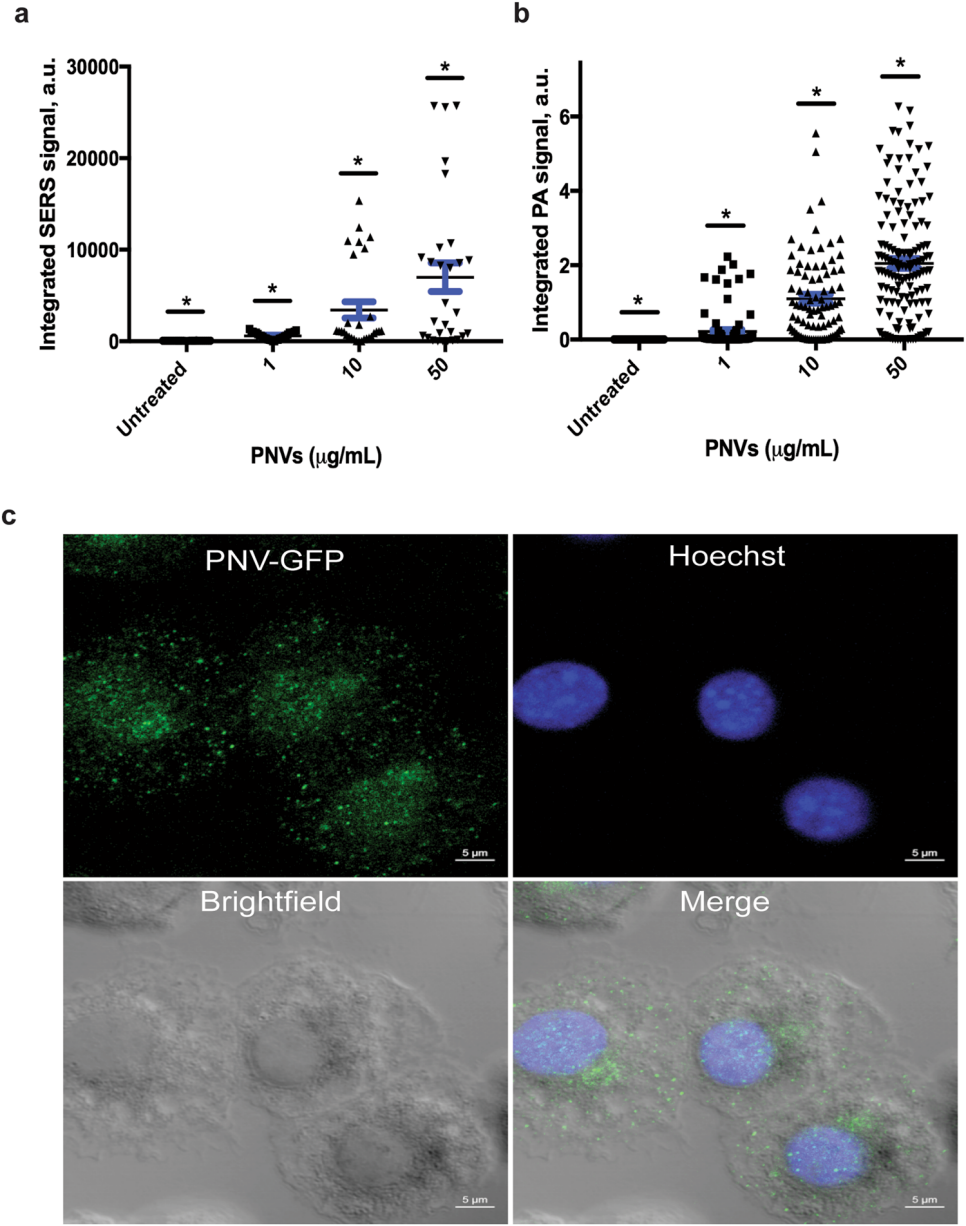
PNVs are taken up by DCs in a concentration-dependent manner. DCs were untreated or treated with 1, 10, and 50 μg/ml of PNVs for 24 hours and assessed by (**a**) Raman and (**b**) PAM. For all data, each symbol—circle (untreated), square (1 μg/ml PNVs), triangle (10 μg/ml PNVs), or up-side-down triangle (50 μg/ml PNVs)—corresponds to either the integrated SERS signal or integrated PA signal from an individual cell. The horizontal blue line represents the mean of the standard error, *p ≤ 0.05 as determined by Anova using Bonferroni correction. The PAM data are representative of 2 independent experiments, with a combined minimum of n = 87. The Raman data are representative of one experiment; n = 30. (**c**) Confocal microscopy images of DCs treated with 50 μg/ml PNV-GFP overnight, that were washed, fixed, and stained with anti-GFP followed by goat anti-rabbit Alexa Fluor 488 conjugated antibody and the nuclear dye Hoechst; upper left = PNV-GFP, upper right = Hoechst, lower left = bright-field, lower right = merged.

SERS signals from each sample were collected with a Raman spectrometer (Horiba Jobin Yvon LabRam HR800, Edison, New Jersey) according to the specifications and methods reported by Nima *et al*.^30^. Briefly, the DCs were either untreated or treated with 1, 10, and 50 µg/mL of PNVs for 24 hours at 5% CO_2_, 37 °C, 100% humidity. Afterwards, the cells were washed and fixed with 4% paraformaldehyde for 20 minutes at room temperature followed by several wash steps: 1X PBS repeated 3 times and with deionized water 5 times. Subsequently, randomly selected individual cells were “scanned” or “mapped;” the edges of the mapping were defined by the cytoplasmic boundary of the cell itself (with an average square size mapping of 16 µm × 16 µm). There was no overlap in the cells chosen for Raman mapping because each cell was mapped individually and no cells were mapped twice. The Raman spectrometer was equipped with an He-Ne laser (785 nm) and an Olympus BX-51 lens with 100x micro-objective connected to a CCD camera. The spectra were collected using 600-line/mm gratings and equal acquisition times. The spectrometer featured a 3D (x-y-z) auto-adjustable stage to map the scanning of a specific area at a minimum distance of 1 μm. For all measurements, the instrument was calibrated using the Si-Si Raman signal, located at a 521 cm^−1^ Raman shift. SERS spectra were collected in DuoScan® mode by scanning 10 individual cells, for a total acquisition of 30 spectra per treatment condition per time of incubation. Each spectrum represents the SERS signal collected from a full-size cell, around a 16 µm × 16 µm (x-y) area. Raman spectra was acquired between 1000 and 1100 cm^−1^, where the relative peak of PATP at 1080 cm^−1^ was present. Each spectrum was recorded, baselined, and background-corrected from the 1080 cm^−1^ peak intensity of the PATP detected using LapSpec® software. SERS data are shown in Fig. 2a as integrated SERS signals recorded in function of the different concentration of PNVs incubated with the cells.

### Laser Scanning Photoacoustic Microscopy (PAM)

The slides for PAM were prepared as described for Raman (see above). A custom laser scanning PAM was developed based on the Olympus IX81 inverted microscope platform. XY galvo mirrors (GVSM002, Throlabs Inc., Newton, NJ) steered a 532-nm laser beam (LUCE 532, Bright Solutions, Italy) with the microscope via a single-mode optical fiber. The laser beam was delivered to the sample from the bottom by a 10x objective (DPlan 10x, Olympus Inc.). The acoustic waves were acquired by a focused transducer (V316, 20 MHz, 12 mm focal distance, Olympus-NDT Inc.) fixed over the sample (transmission configuration). To provide acoustic coupling between cells on the glass slides and the transducer, a custom cup (made of a 3/4-inch disposable plastic weighing dish) was attached to the glass slide using epoxy glue and filled with deionized water. Signals from the transducer were amplified by a 20-dB amplifier (0.05–100 MHz bandwidth, AH-2010–100, Onda Corp) and recorded by a PC equipped with a high-speed digitizer (PCI-5124, 12-bit card, 128 MB of memory, National Instruments, Austin, TX). System synchronization and laser triggering were performed by a digital waveform generator (DG4062, Rigol, Beijun, China).

PA images (100 × 100 pixels) were acquired from the 200 × 200 µm sample area. Laser beam diameter spot size was estimated to be ~2.3 µm (FWHM), and laser scan step was 2 µm. The pitch separating these points was done so that the laser spots overlapped only slightly (~5%). For each sample point, 40 PA signals were averaged and maximal amplitude of the acoustic wave was recorded. Optical microscopy images were collected by a DP72 camera (Olympus Inc.) using a custom ring illuminator mounted on the transducer. Both optical and PA images were processed using a custom ImageJ macro to manually identify individual cells and integrate the PA signals from all the pixels corresponding to these regions. Typically, each PA image contained 5–10 cells. Figure 2 shows integrated PA signals for the individual cells.

PA imaging was performed in high-resolution mode on the same setup, using 100x Plan Fluor focusing objective, which provided tight laser beam focusing that resulted in lateral and axial imaging resolution of 300 and 900 nm, respectively. 100 × 100 pixel PA images were acquired from the sample area of 20 × 20 µm with a 0.2 µm scan step. Multiple 2D PA images were acquired along the vertical microscope axis by displacing the focusing objective in axial direction with a 1-µm step to create a 3D model of the sample absorption.

### Transmission Electron Microscopy (TEM)

1 × 10^6^ DCs were seeded in 35-mm dishes one day prior to treatment. Cells were untreated or treated with 1 and 10 µg/ml PNVs and incubated for 24 hours before fixation. The samples were then prepared for TEM as previously described by Majeed *et al*.^39^. Briefly, the DC samples were fixed with 2.5% glutaraldehyde in 0.1 M sodium cacodylate buffer for 20 minutes at 4 °C. Subsequently, the samples were washed 3 times with 0.1 M sodium cacodylate buffer at 5-minute intervals. The samples were post-fixed with 1% osmium tetroxide, 0.8% potassium ferricyanide in 0.1 M sodium cacodylate buffer for 1 hour at room temperature in the dark. The samples were then washed with 0.1 M sodium cacodylate buffer 3 times at 5-minute intervals and stained with 1% tannic acid for 20 minutes on ice, followed by staining with 0.5% uranyl acetate for 1 hour at room temperature. Next, the samples were dehydrated with a graded ethanol series and embedded in epoxy resin. Afterwards, 70 nm sections were cut with a diamond knife on a Leica UltraCut7-UCT microtome and post-stained with 1% uranyl acetate and Reynold’s lead citrate (Electron Microscopy Sciences) before being viewed. A JEOL JEM-2100F TEM with the field emission gun and EDAX EDS system option was used to obtain the images of both the PNVs and the DCs. The prepared DC samples were imaged at 80 kV. Prior to TEM imaging, all cell samples were coated with ~3-nm-thick carbon films to improve thermal and electrical conductivity.

### Confocal Microscopy

The DCs were treated with 50 μg/ml PNV-GFP for 24 hours in a tissue culture incubator at 5% CO_2_, 37 °C, 100% humidity. They were then washed and fixed with 4% paraformaldehyde for 10 minutes at room temperature. Next, the DCs were permeabilized with 0.5% saponin for 15 minutes, then stained for anti-GFP (Novus Biologicals, NB600-308) overnight at 4 °C. Afterwards, the cells were washed and stained with goat anti-rabbit Alexa Fluor 488 conjugated antibody (Jackson ImmunoResearch Laboratories, 111-546-144) for 1 hour at room temperature, followed by nuclear staining with Hoechst dye for 10 minutes. Washed slides were then mounted and imaged with a Zeiss confocal microscope (Digital Microscopy Core, UAMS).

### Statistical Analysis

All experiments were performed in triplicates with at least technical duplicates in each experiment. Data are reported as standard error of the mean, *p ≤ 0.05, as determined by Anova using Bonferroni correction.

## Results

### The PNVs associated with the DCs

In order to demonstrate that the PNVs can be potentially used as a carrier for cancer vaccines, DCs must be able to associate and internalize them. The architecture of the our PNVs consists of AuNRs covered by 1–2 nm of Ag, decorated with the strongly SERS-active scattering molecule PATP, and ultimately encapsulated by a PEG layer for better solubility in aqueous solutions and further biofunctionalization capability (Fig. 1b,c)^31^. The PEG layer also protects the Ag from oxidation and possible leakage, which could impact cell viability^40^. We confirmed the size distribution of the PNVs by AFM. As shown, both height and phase images reveal that the PNVs were mostly uniform in size and that they corresponded to the AR = 3.0 ± 0.23 as previously described (Fig. 1d,e and Supplementary Fig. 1a–c)^30^. To assess if the PNVs could be internalized, DCs were untreated or exposed to 1, 10, and 50 μg/ml of the PNVs for 24 hours, and then analyzed by Raman spectroscopy and PA microscopy. Individual DCs were randomly selected, and cells were scanned, as described before ^30^. The intensity of the SERS and PA signals collected from the DCs exposed to the PNVs, compared to that of the untreated controls, indicated a concentration-dependent increase in the amount of PNVs that could be taken up by the cells (Fig. 2a and 2b).

In order to further validate that the PNVs accumulated within the cells, we performed high-resolution PAM^23, 41–43^. PAM confirmed that PNVs accumulated intracellularly (Supplementary Fig. 2). PA signals corresponding to the PNVs mostly originated from the cell cytoplasm. There were no particles collocated within regions corresponding to the cell nucleus (DAPI stain), as evident by the PNVs’ diffused (red) appearance within the DC compartment (Supplementary Fig. 2) and the absence of the PNV signal in the cell nucleus region (blue, Supplementary Video 1). To verify the PNVs’ accumulation in the intracellular compartments of DCs, PNVs were conjugated with GFP to allow detection of the resulting PNV-GFP by immunofluorescence. DCs were treated with 50 μg/ml PNV-GFP for 24 hours, fixed, and stained with anti-GFP, and subsequenlty stained with a secondary antibody, goat anti-rabbit Alexa Fluor 488. According to the immunofluorescence results, the PNV-GFP were internalized in the DCs (Fig. 2c), confirming the results of the SERS and PAM imaging. PNV-GFP were diffused and distributed in nearly all parts of the DC, reflective of the DCs’ phagocytic properties.

In summary, Raman, PAM, and confocal microscopy results indicated that the PNVs can be located within and around the DCs. However, it is possible that some of the PNVs could be located on the surface membrane of the DCs, which may account for the increase in surface staining associated with them.

### PNVs are non-cytotoxic to DCs

**Figure 3:**
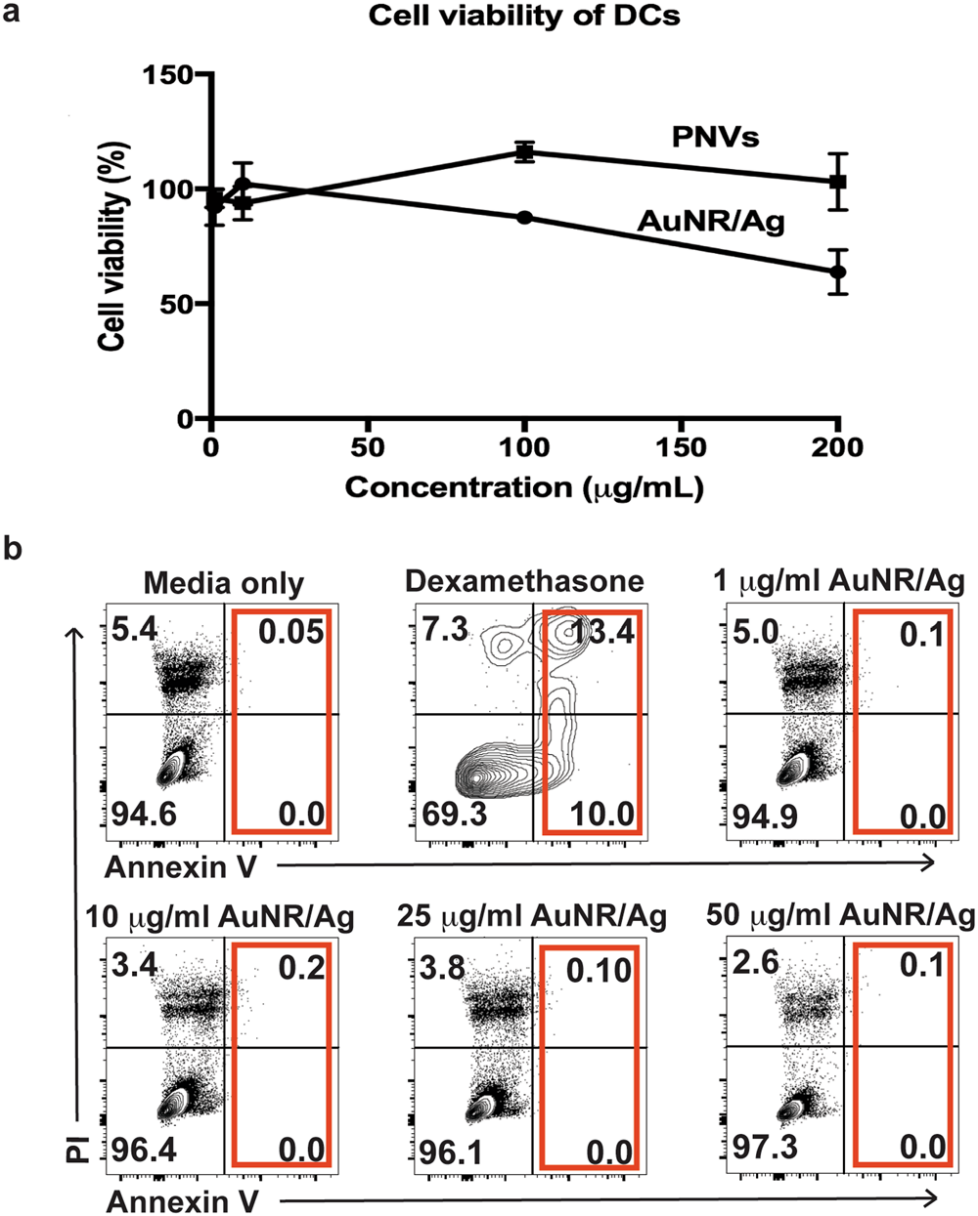
PNVs are non-cytotoxic up to the 200 μg/ml concentration. (**a**) DCs were treated with 1, 10, 100, and 200 μg/ml of PNVs or AuNR/Ag for 24 hours, and viability was assessed by measuring mitochondrial function using WST-1. Combined data represent 3 independent experiments. (**b**) DCs were treated with 1, 10, 25, and 50 μg/ml of AuNR/Ag, with medium alone, or with dexamethasone (400 μM as positive control) for 5 days and assessed by Annexin V staining, which measures the number of apoptotic events, as indicated in the red box. The combined data represent 2 independent experiments.

To demonstrate that PNVs could be used as a vaccine carrier in DC-based applications, we examined whether PNVs could affect DC viability. We cultured DCs with 1, 10, 100, and 200 μg/ml of the base vector, AuNR/Ag, or the PNVs for 24 hours and analyzed the results with a WST-1 assay. In contrast to AuNR/Ag, the PNVs had no effect on cell viability, even at the highest treatment concentration studied of 200 μg/ml (Fig. 3a). Treatment with concentrations greater than 50 μg/ml of AuNR/Ag did influence viability (70% +/−10%), but the affected cells were not statistically different from the PNV-treated cells (Fig. 3a). To further examine the potential cytotoxic nature of the base vector, AuNR/Ag, on DCs, we assessed whether treatment time would affect DC apoptosis. We treated DCs with 1, 10, 25, and 50 μg/mL of AuNR/Ag for 5 days. As assessed by Annexin V staining, AuNR/Ag up to the 50 μg/ml concentration did not induce any discernable apoptosis when compared to medium-only control and the dexamethasone-positive controls (Fig. 3b). These experiments show that up to the 50 μg/ml concentration, DCs are unaffected by both the PNVs and AuNR/Ag treatment, with viability remaining near 100%.

### PNVs localize within DC endosomes

**Figure 4:**
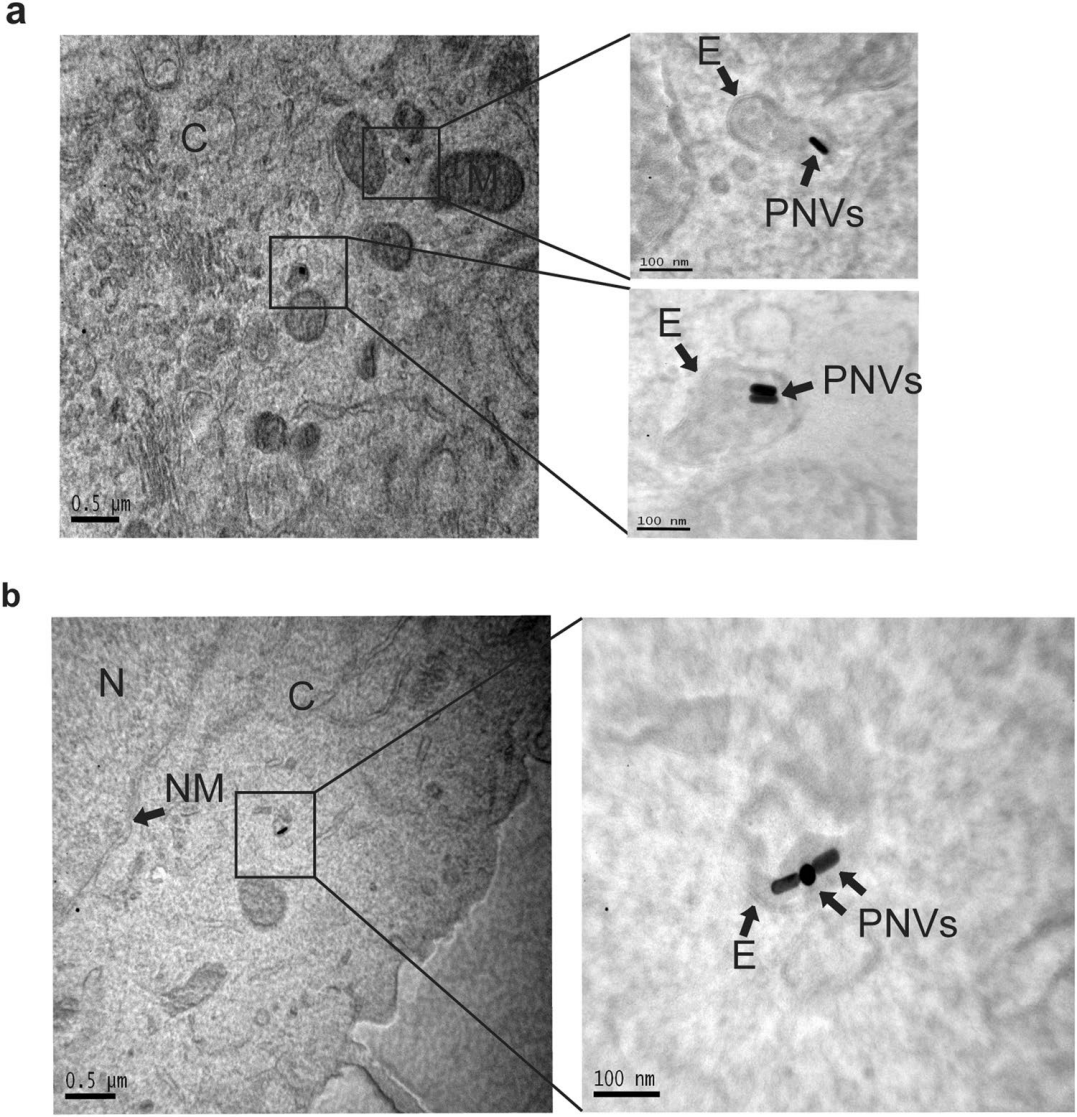
PNVs can be found within intracellular compartments of the DCs. DCs were untreated or treated with 1 and 10 μg/ml of the PNVs for 24 hours, then the samples were prepared and assessed by TEM. The images are of DCs. (**a**) 10 μg/ml PNVs treated and (**b**) 1 μg/ml PNVs treated, where N = nucleus, NM = nuclear membrane, C = cytoplasm, E = endosomes, and PNVs = plasmonic nano vectors.

Phagocytosis is a specialized form of endocytosis that is carried out by DCs^44–46^. If the PNVs are actively being phagocytized, they will accumulate in intracellular compartments such as endosomes. To demonstrate this, DCs were treated with 1 μg/ml or 10 μg/ml of the PNVs for 24 hours, and then examined by TEM. The results showed that PNVs were enclosed within the endosomes of the DCs (Fig. 4a,b), indicating that PNVs were actively phagocytized and compartmentalized. We observed that PNVs in endosomes were either single nanorods or clustered in pairs or triplicates (Fig. 4a,b, Supplementary Figure 3, and Supplementary Figure 4). However, given the location and the specific nature of the TEM analysis, we cannot completely dismiss the possibility that larger clusters are not present in some cells, though we did not observe this in the cells that were imaged.

### Phagocytosis of PNVs induced DC maturation

As an indirect measurement of phagocytosis, we evaluated the surface expression of DEC-205 (CD205), an endocytic marker expressed on DCs^47–49^. DCs were treated with 1, 10, and 50 μg/ml of the PNVs for 3 and 7 days. Day 0 (D 0) cells served as the untreated controls, while lipopolysaccharide (LPS)-treated DCs were the positive controls. It should be noted that LPS-induced changes in receptor expression are used solely as a guideline, and, thus, direct comparison between the LPS- and PNV-induced changes in receptor expression should be done with caution. This is because LPS is a known adjuvant of DCs and binds to Toll-like receptor 4 (TLR4), found on the surface of DCs. Signaling through TLR4 has been shown to be critical in the activation and maturation of DCs. In essence, we sought to see whether PNVs can turn on these receptors in comparison to untreated controls, and are less concerned with whether receptors are turned on to the same extent as LPS.

**Figure 5:**
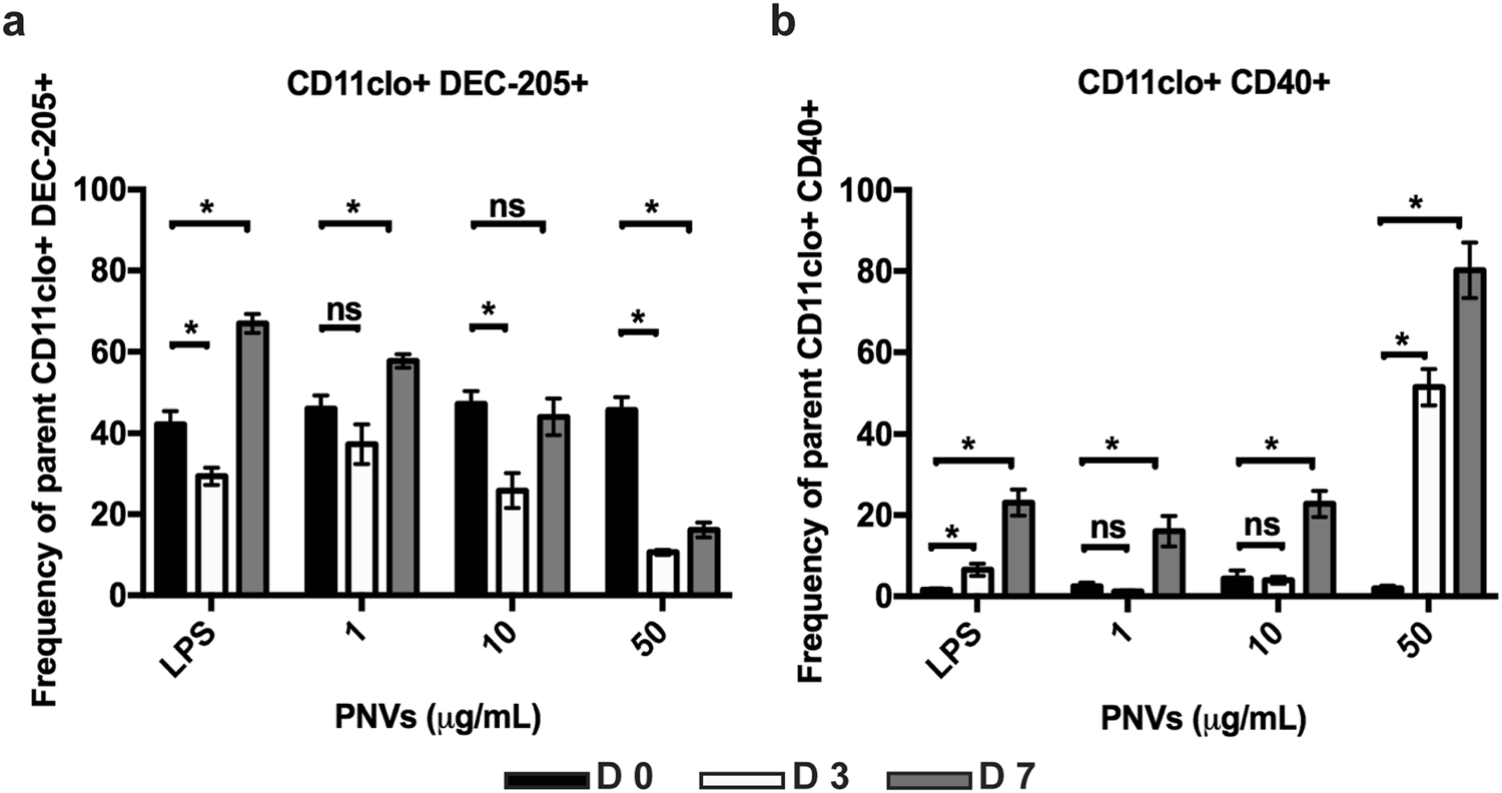
PNVs caused a down-regulation of the endocytic DEC-205 receptor and an up-regulation of the CD40 receptor. DCs were treated with 1, 10, and 50 μg/ml of PNVs or LPS (5 μg/ml) as a positive control, and flow cytometry was performed for (**a**) DEC-205 and (**b**) CD40 receptor expression. Day 0 (D 0) = untreated controls, black filled bars, Day 3 (D 3) = white non-filled bars, and Day 7 (D 7) = gray filled bars. These experiments are a compilation of 3 independent experiments; n = 3 per group.

On D 3, PNV-treated DCs were able to down-regulate expression of the DEC-205 receptor on the cell surface, —similar to LPS-positive controls, —indicating that PNVs were being internalized by DCs (Fig. 5a). The lowest concentration of the PNVs, 1 μg/ml, appeared to cause the down-regulation of DEC-205 on the cell surface, though this was found to be non-significant compared to D 0 controls (Fig. 5a). However, treatment with higher concentrations of PNVs induced increased down-regulation of DEC-205, coinciding with the increase in levels observed in the Raman and PAM data (Fig. 2a,b). By D 7, DEC-205 receptor levels had markedly increased from that those observed on D 3 (Fig. 5a). Interestingly, we found that this increase correlated with the up-regulation of CD40, a marker of DC maturation (Fig. 5b). Additionally, by D 7, higher concentrations of PNVs had increased the expression of CD40, compared to untreated D0 controls (Fig. 5b).

**Figure 6:**
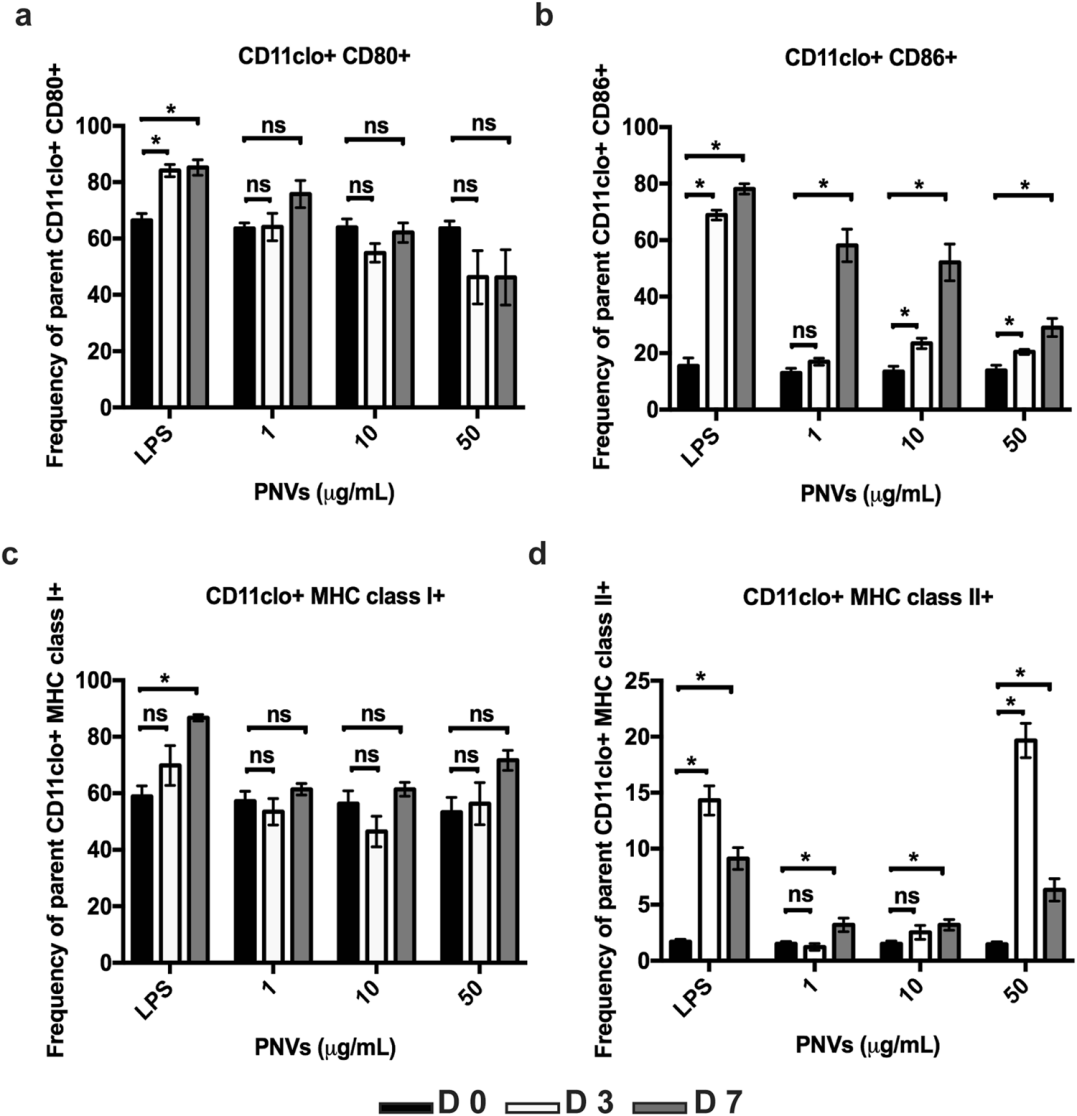
PNVs induced receptor expression on the surface of DCs, indicative of their state of activation and maturation. DCs were treated with 1, 10, and 50 μg/ml of PNVs or with LPS (5 μg/ml); harvested at Day 0 (D 0 untreated controls, black filled bars), Day 3 (D 3, white non-filled bars), and Day 7 (D 7, gray filled bars); and assessed for receptor expression by flow cytometry for expression of (**a**) CD80, (**b**) CD86, (**c**) MHC class I, and (**d**) MHC class II. These experiments are a compilation of 3 independent experiments; n = 3 per group.

Because PNV treatment caused an increase in the expression of CD40 on the cell surface of DCs, we investigated whether other surface markers of maturation also increased. We examined key markers in DC activation and maturation: CD80, CD86, MHC class I, and MHC class II. As expected, as early as D 3 and as late as D 7, LPS-treated positive controls were able to increase expression of these receptors: CD80, CD86, MHC class I and MHC class II (Fig. 6a–d). Upon examination of CD80 receptor expression after DCs were treated with 1, 10, and 50 μg/ml of PNVs, we found that no concentration of PNVs induced the CD80 receptor (Fig. 6a). However, the CD86 receptor was induced by the 10 and 50 μg/ml PNV concentrations (Fig. 6b). The lowest concentration of 1 μg/ml PNV induced the expression of the CD86 receptor but this was delayed to D 7 (Fig. 6b). For MHC class I, DCs treated with 1, 10, and 50 μg/ml of PNVs were unable to induce their expression (Fig. 6c). In contrast, the MHC class II receptor was induced at the 50 μg/ml PNV concentration by D 3 but was delayed at the 1 and 10 μg/ml PNV concentrations until D 7 (Fig. 6d).

In summary, PNV concentrations of 1, 10, and 50 μg/ml induced CD40, CD86, and MHC class II expression. However, at the 1 μg/ml PNV concentration, receptor expression was delayed until D 7. Though delayed, the PNVs followed the same trend of turning on some of the receptors similar to the LPS controls. While the LPS controls induced expression more efficiently than the PNV-treated DCs, LPS’ mechanisms of action are different than those of PNVs. However, as mentioned before, direct comparison between the two treatments should be cautioned due to the highly LPS-sensitive nature of TLR4. It is not known if PNVs can bind TLR4 or any other TLRs. In general, DCs treated with 1, 10, and 50 μg/ml of the PNVs were able to increase the expression of CD86 compared to CD80 (Fig. 6a,b) and of MHC class II compared to MHC class I (Fig. 6c,d).

## Discussion

In this study, we used PAM, SERS, and confocal microscopy to demonstrate that our PNVs adhere to and are taken up by DCs. Additionally, TEM indicated that PNVs are taken up and shuttled into the endosomes. This specific uptake and location suggests that PNVs could be cross-presented, a process that has been shown to induce a robust anti-tumor response^50–52^. However, more detailed and functional studies are needed to investigate this fully. Moreover, we found that the PNVs do not affect DC viability up to the highest tested concentration of 200 μg/ml, and, no significant change in DC cell apoptosis by AuNR/Ag was observed up to 50 μg/ml. These concentrations are well above the anticipated concentrations needed *in vivo* for proper stimulation of DCs.

Subsequently, we assessed the kinetics and dynamics of the DC receptor expression levels as a function of maturation state. In order to compare and contrast, we used an established natural bacterial adjuvant, LPS, as a control. In general, similar to LPS, we found that PNVs initially down-regulate DEC-205, as seen on day 3. Because DEC-205 is a marker of endocytosis, this complements our microscopy results on the uptake of PNVs. However, by day 7, DEC-205 expression is up-regulated in both the LPS and the 1 μg/ml PNV treated samples. Higher doses of the PNVs (10 and 50 μg/ml) caused prolonged and dose-dependent suppression. Additionally, we found that some of the activation/maturation factors were not affected at the concentrations tested. Namely, MHC Class I and CD80 expression did not significantly change up to day 7 and at the PNV concentration of 50 μg/ml. In contrast, CD40, CD86, and MHC Class II showed dose and time-dependent increases in expression levels. The significance of the PNVs’ ability to induce the expression of some but not the other receptors is very intriguing and warrants further exploration. Overall, the results show that the PNVs may have adjuvant-like capacities, which suggests that they might be able to replace current adjuvants (LPS, Freund’s incomplete adjuvant, and aluminum compounds) or at least lower the amount of adjuvant required in immunotherapy, as even the 1 μg/ml concentration was able to significantly up-regulate MHC class II, CD40, and CD86.

The size, surface charge, shape, and composition of PNVs could influence their internalization and cytotoxicity, as well as the activation/maturation state of the DCs. We have found that PNVs with an aspect ratio of 3 (diameter of 12 nm, and length of 36 nm) can be internalized into DCs, supporting similar work demonstrating that AuNPs between 40–50 nm are much more efficiently internalized than smaller ones (10 nm)^26, 53^. For example, using a macrophage cell line, smaller AuNPs (4 nm) were found to bind the high-mobility group box-1 of TLR9, reducing TLR9 signaling and impairing the production of TNF-α. These results imply that larger NPs are preferred in DC-based immune applications over smaller ones^54^. Surface charge and coating could also play a role in PNV internalization, as surface charge could impact cell-mediated phagocytosis. Furthermore, coating the PNVs with PEG had no effect on cytotoxicity (Fig. 3B). Also, as shown by zeta potential, our PNVs were negatively charged, −50.0 +/−20 mV. Previously it was shown that positively charged AuNPs could enhance internalization. However, this also increased the likelihood of cytotoxicity^55, 56^. Though our PNVs were negatively charged, this appeared to have no influence on the state of apoptosis or viability of the DCs.

In the present investigation, we have established that 1) plasmonic nano vectors (PNVs) can be phagocytized by DCs; 2) PNVs are non-cytotoxic; and 3) PNVs can induce a DC maturation-like phenotype on their own. These findings support the development of PNVs as a more efficient carrier for cancer vaccine delivery. Future studies will focus on the functionalization of PNVs with cancer antigens and multiple DC targeting receptors in tumor mouse models to determine PNVs’ effectiveness as vaccine adjuvants. These results may have significant, positive implications for using PNVs as a carrier for DC-targeted cancer vaccines.

## Electronic supplementary material

Supplemental video 1

Supplementary Figures
