# Supplementary material for: Modifying Dendritic Cell Activation with Plasmonic Nano Vectors
Source: Sci Rep. 2017 Jul 14;7:5513. doi: 10.1038/s41598-017-04459-1 (PMC5511287; doi:10.1038/s41598-017-04459-1)
Supplement: Supplementary file 2 — Supplementary Figures [file 41598_2017_4459_MOESM2_ESM.pdf]

Modifying Dendritic Cell Activation with Plasmonic Nano Vectors

Kieng Bao Vang[$]*, Ingrid Safina[$], Emilie Darrigues[$], Dmitry Nedosekin[#], Zeid A. Nima[$], Waqar Majeed[$], Fumiya Watanabe[$], Ganesh Kannarpady[$], Rajshekhar A. Kore[§], Daniel Casciano[$], Vladimir Zharov[#], Robert J. Griffin[§], Ruud P. M. Dings[§], and Alexandru S. Biris[$]

Supplementary Figure 1

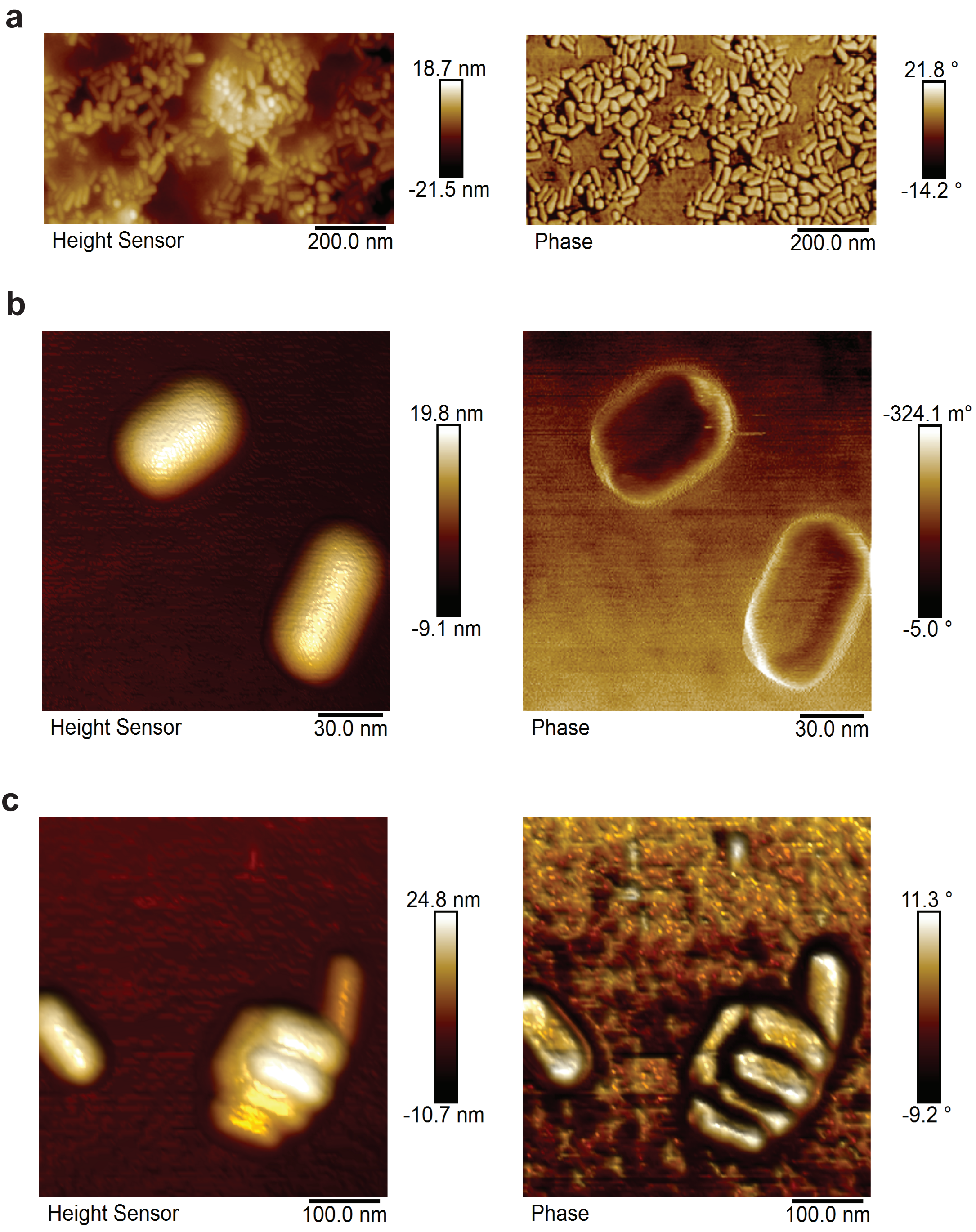

Supplementary Figure 2

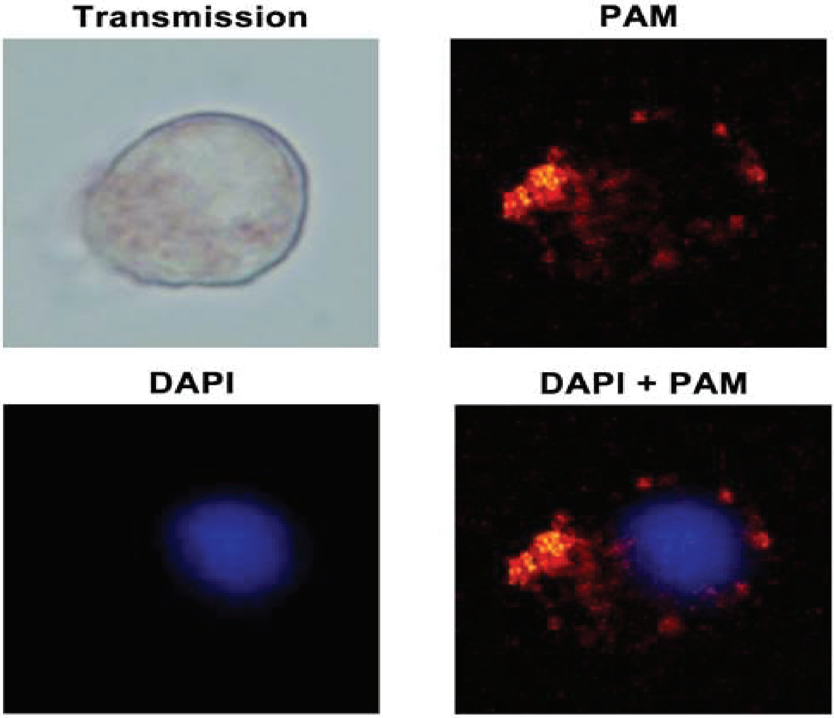

Supplementary Figure 3

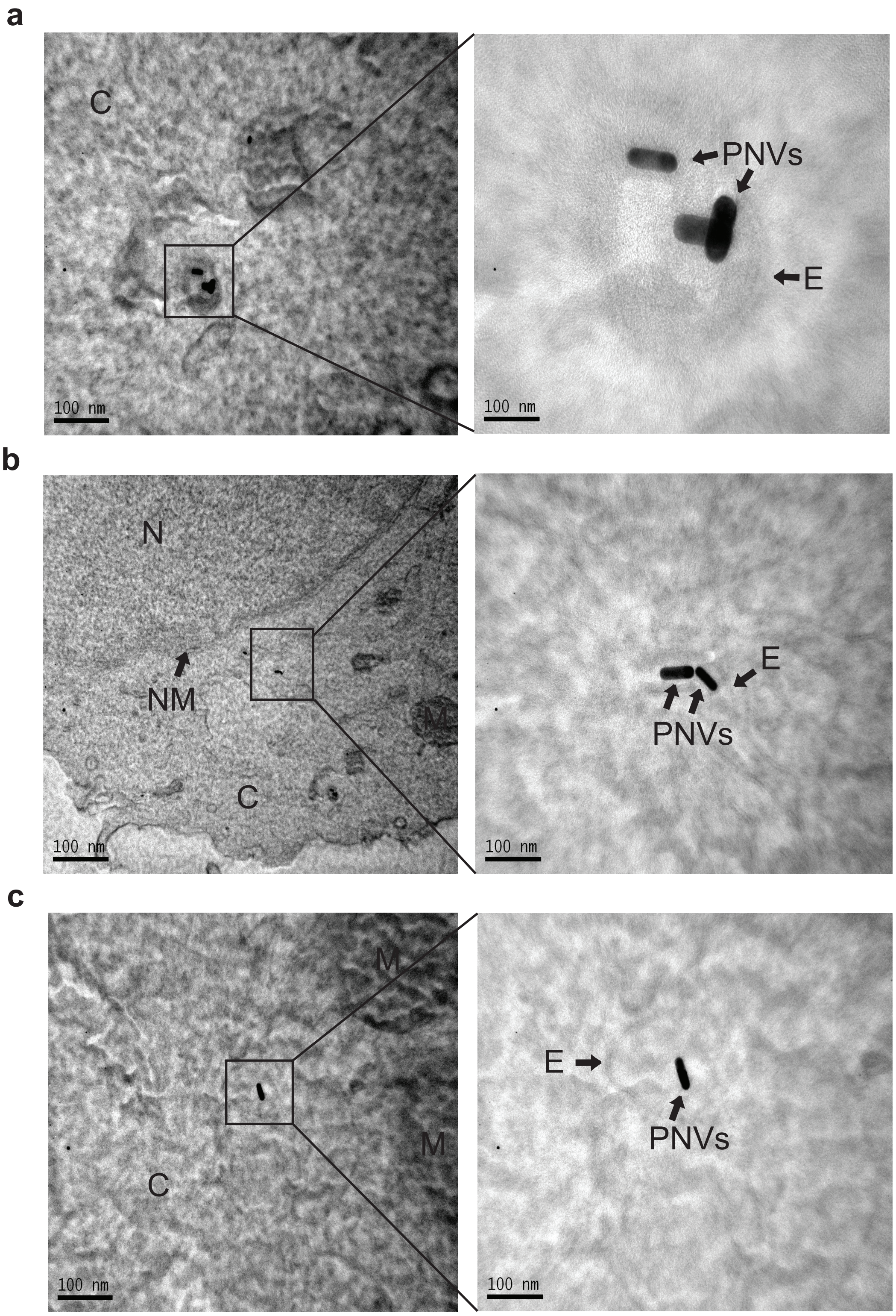

Supplementary Figure 4

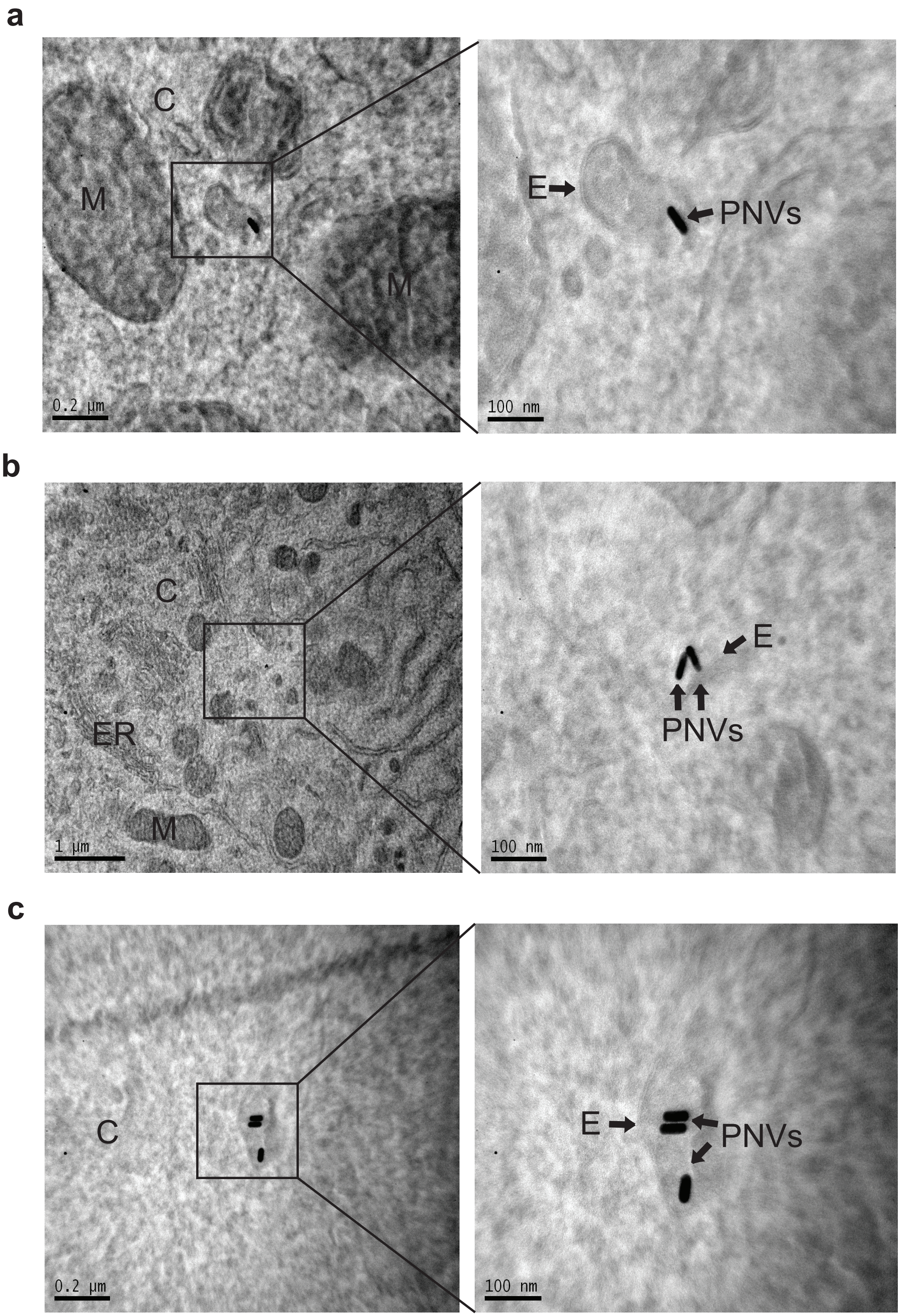

Supplemental Figure 1

AFM images of the PNVs at different scanning scales as shown in **a., b.,** and **c.** Left side columns are images of the scanned height view of the PNVs, and right side columns are images of the phase view of the PNVs derived from the height images.

Supplemental Figure 2

High-resolution PA images of DCs treated with 1 μg/ml of the PNVs for 24 hours. Transmission (upper left), the PNVs are depicted in red (PAM, upper right), DAPI (blue, bottom left) and DAPI+PAM (merge, bottom right). The images were acquired with a laser wavelength of 532 nm and a pulse energy of 300 nJ, averaging 10 signals/pixel; PA images are 100×100 pixels.

Supplemental Figure 3

The PNVs are found within intracellular compartments of the DCs. DCs were treated with 1 μg/ml of the PNVs for 24 hours. Samples were prepared as described in the methods and then assessed by TEM. N = nucleus, NM = nuclear membrane, M = mitochondria, C = cytoplasm, E = endosomes, and PNVs = plasmonic nano vectors.

Supplemental Figure 4

The PNVs are found within intracellular compartments of the DCs. DCs were treated with 10 μg/ml of the PNVs for 24 hours. Samples were prepared as described in the methods and then assessed by TEM. N = nucleus, NM = nuclear membrane, M = mitochondria, C = cytoplasm, E = endosomes, and PNVs = plasmonic nano vectors.

Supplemental video 1

3D video of DCs treated with 1 μg/ml of the PNVs for 24 hours. The PNVs are indicated in red and the blue color shows DAPI staining of the nuclei. Stack of 17 PA/fluorescence images were acquired with 1 μm displacement of a focusing objective.
